# Very Low Volume Sprint Interval Exercise Suppresses Subjective Appetite, Lowers Acylated Ghrelin, and Elevates GLP-1 in Overweight Individuals: A Pilot Study

**DOI:** 10.3390/nu9040362

**Published:** 2017-04-05

**Authors:** Adrian Holliday, Andrew K. Blannin

**Affiliations:** 1 School of Sport, Exercise and Rehabilitation Sciences, University of Birmingham, Birmingham B15 2TT, UK; a.k.blannin@bham.ac.uk; 2 Institute for Sport, Physical Activity and Leisure, Leeds Beckett University, Leeds LS6 3QS, UK

**Keywords:** high-intensity intermittent exercise, food intake, satiety, hunger, appetite hormones

## Abstract

High-intensity exercise has been shown to elicit a transient suppression of appetite and create a more anorexigenic profile of appetite-associated hormones. It is yet to be fully elucidated whether such a response is observed following very low-volume, intermittent exercise at supramaximal intensity in those who are overweight. Eight overweight individuals (BMI 27.7 ± 1.7 kg·m^2^) completed resting (REST) and exercise (EX) trials in a counterbalanced order. EX consisted of 4 × 30 s “flat-out” cycling on an ergometer (adapted Wingate test). Two hours post-exercise (or REST), participants were presented with an *ad libitum* meal. Subjective appetite measures and blood samples were obtained throughout. Subjective appetite, measured using VAS, was significantly lower immediately after exercise compared with REST (38.0 ± 28.5 mm vs. 75.1 ± 26.2 mm, *p* = 0.018, *d* = 1.09). This difference remained significant 30 min post-exercise. Acylated ghrelin concentration was suppressed in EX compared with REST immediately post-exercise (113.4 ± 43.0 pg·mL^−1^ vs. 189.2 ± 91.8 pg·mL^−1^, *p* = 0.03, *d* = 1.07) and remained lower until the *ad libitum* test-meal. Area-under-the-curve for GLP-1 concentration was significantly greater for EX, versus REST. There was no difference in absolute *ad* *libitum* intake or relative energy intake. As little as 4 × 30 s of “flat-out” cycling was sufficient to elicit a transient suppression of appetite and an enduring suppression of plasma acylated ghrelin. Nonetheless, food intake 2-h post-exercise was unaffected.

## 1. Introduction

It has been repeatedly shown that high-intensity, continuous aerobic exercise elicits a transient suppression of appetite in lean, recreationally active individuals [1,2,3,4,5,6,7]. As the intensity dependency of this appetite response appears well founded, and if the response is exclusively driven by intensity and is independent of duration and energy expenditure, it may be possible to elicit a suppression of appetite with very low-volume bouts of supramaxial exercise. High-intensity interval exercise (HIE) and high-intensity training (HIT) have been shown to elicit physiological responses [8,9] and adaptations [10,11,12] akin to that seen with traditional, longer duration, continuous aerobic exercise. Consequent improvements in metabolic health, such as acute [13,14] and chronic improvements in glycaemic control [15,16], and acute attenuation of postprandial lipaemia [17,18], are likely to be of particular relevance for those who are overweight and obese. However, one criticism often levelled at such low-volume exercise bouts is a low energy cost. It is often argued that HIT is an ineffective form of exercise for inducing weight-loss in overweight and obese individuals, as the low energy cost is unlikely to yield an energy deficit sufficient to promote weight-loss. As a result, despite evidence for the effectiveness of HIE for improving health outcomes such as insulin insensitivity and blood dyslipidemia amongst overweight and obese individuals, there is contention regarding the recommendation of such exercise for this population [19,20,21].

While this claim is based largely on energy kinetics, appetite responses to HIE must also be considered. An initial investigation in recreationally active individuals [22] demonstrated that 6 × 30 s Wingate tests transiently suppressed appetite. However, food intake at an *ad libitum* buffet meal was no different to that of a control condition. Immediate post-exercise appetite suppression has also been observed after just 4 × 30 s “all out” sprint on a treadmill in healthy weight males [23], although a reduced-volume bout of 10 min of cycling at 60W interspersed with 2 × 20 s maximal effort sprint did not suppress appetite in healthy males [24]. Appetite responses to HIE may be mediated by anorexigenic changes in circulating concentrations of the hunger hormone acylated ghrelin [22]. Contention remains regarding responses in the satiety peptide glucagon-like peptide 1 (GLP-1); non-significant, modest suppressions in GLP-1 have been accompanied by appetite suppression [25], while more profound suppressions have been seen in the absence of changes in appetite [26]. 

When investigating appetite responses to HIE in overweight and obese individuals, similar appetite suppression was observed after high-intensity intermittent cycling, compared with isocaloric moderate-intensity continuous cycling [27,28]. This was despite a more anorexigenic profile of plasma GLP-1 [28]. However, in both of these studies, the high-intensity intermittent exercise bout was of submaximal intensity. Male participants in the study of Alkahtani et al. [27] performed exercise for 15 s intervals at 85% VO_2peak_, separated by 15 s of active recovery at 0 watts, while the male and female participants in the study of Martins et al. [28] completed intervals of 8 s at high-intensity with 12 s of low-intensity active recovery to elicit a heart rate of approximately 85% maximal heart rate. Utilising a more strenuous exercise protocol, Sim et al. [29] observed a reduction in *ad libitum* food intake in overweight men after 15 s bouts of cycling at 170% VO2_max_, which was not seen with energy matched submaximal intermittent bouts. The appetite response to maximal exercise in overweight and obese individuals remains unclear, particularly in females.

The aim of this pilot study was to investigate the effect of very low-volume “flat out” sprint interval cycling on appetite, food intake, and the circulating concentration of the appetite-regulating hormones acylated ghrelin and GLP-1 in overweight individuals. Not only will this shed further light on appetite response, but it will also assess the feasibility of such bouts for overweight men and women. It was hypothesised that 4 × 30 s maximal effort cycling would induce a suppression of subjective appetite, and that this would be associated with an anorexogenic change in hormones.

## 2. Materials and Methods

### 2.1. Participants 

Twelve overweight, low- to moderate-activity individuals were recruited, principally from the University of Birmingham’s student and staff body. Inclusion criteria were: a BMI of 25.0–34.9 kg·m^−2^, a score of ≤3000 MET-minutes per week on the International Physical Activity Questionnaire (IPAQ), currently weight-stable, not attempting weight-loss, and not currently undertaking any form of vigorous exercise. Exclusion criteria were: resting blood pressure of >140/90 mm·Hg, irregular cardiac activity at rest (as assessed with the use of a resting ECG), illness such as upper respiratory tract infections, smoking, and the prescription or taking of medication likely to affect appetite or induce weight-loss (participants were asked to disclose any medication that they had been prescribed or were currently taking; no disclosures were made by any participant). After written and verbal information regarding the nature of the study and any associated risks were provided, written consent was obtained. Of the twelve participants recruited, three withdrew and one was excluded from the results on the basis of their resting acylated ghrelin concentration, which was >2 SD from the mean [30]. Therefore, eight participants completed the study (four males, four females; mean age = 34 ± 12 years; BMI = 27.7 ± 1.7 kg·m^−2^, Dutch Eating Behaviour Questionnaire (DEBQ, [31]) score for restraint = 2.7 ± 0.8; IPAQ score 1752 ± 770 METS·min·week^−1^).

### 2.2. Study Design

A within-subject, counterbalanced, crossover study design was utilised. Participants were randomly assigned to both the exercise (EX) and resting (REST) trial conditions. The study was conducted in accordance with the Declaration of Helsinki. Ethical approval was obtained from the Ethics Subcommittee of the School of Sport, Exercise and Rehabilitation Sciences, University of Birmingham (Application code: ERN_12-0947).

### 2.3. Pre-Testing

A single session of pre-testing preceded the study protocol. Participants reported to the Human Performance Laboratory, in the School of Sport, Exercise and Rehabilitation Sciences, University of Birmingham, and completed a health questionnaire, as a means of pursuing a health screening procedure, and the DEBQ [31], which was used to assess eating behaviour. Height and weight were recorded, before the participant rested in a seated position for a period of 10 min, prior to the measure of resting blood pressure. Upon the completion of this measure, a 10-lead resting ECG was conducted. The ECG trace was recorded and assessed for any abnormality in cardiac activity by a qualified physician. A short familiarisation bout of exercise was then undertaken. Participants completed a single modified Wingate anaerobic power test, as described below in the *Procedures and Protocol* section. The session concluded with the administering of a food diary and instructions on how to accurately record dietary intake for the 24 h period prior to the participant’s first trial. It was made clear that they would be expected to replicate this dietary intake for the 24 h period prior to their second trial.

### 2.4. Procedure and Protocol

After a minimum period of three days after pre-testing, participants returned to the laboratory at approximately 08:00, after an overnight fast, for the first of two experimental trials. Upon arrival, the participants were weighed (weight was recorded at each visit to ensure that participants were weight-stable throughout) and a baseline appetite measure was taken. Participants were then provided with a standardised breakfast meal (two slices of toast (Thick slice, 50/50 bread, ~90 g), margarine (~16 g), and jam (mixed fruit, ~30 g), with a choice of orange or apple juice (~200 mL)), the approximate energy content of which was 415 kcal (71% energy from carbohydrate, 19% from fat, and 10% protein), based on the addition of jam and the selection of orange juice. Once the breakfast was consumed (*t* = 0), the participant began a two-hour rest period before the exercise bout commenced. During this time, the participant remained sedentary within the laboratory, leaving them free to watch television, read, or use a computer. At *t* = 60 min, a second appetite measure was obtained. At *t* = 90 min, a venous cannula was inserted into the anticubital vein of the arm. At *t* = 120 min, an appetite measure was recorded and a resting blood sample was obtained. In the exercise trial condition, the exercise bout commenced immediately. 

The exercise bout consisted of 4 × 30 s “flat out” sprint on a cycle ergometer (Lode Excalibur Sport, Groningen, Netherlands), utilising a modified Wingate anaerobic power test (after conducting pilot testing to assess the feasibility of sprint interval cycling in inactive, overweight individuals, torque factors of 0.6 for males and 0.5 for females were used to allow completion of the 30 s bout). The first bout was preceded by a brief 3 min warm-up period, at a constant load of 40 watts; the first sprint was initiated immediately upon reaching the end of the 3 min period. The four sprint bouts were separated by a 3 min recovery period, during which the power of the cycle ergometer was a constant 40 watts. During this recovery period, the participants were encouraged to remain on the bike, being free to rest or partake in light cycling to facilitate active recovery. The heart rate was recorded throughout the bout, using a heart rate monitor (Polar S625X; Polar Electro Oy, Kempele, Finland), and noted at the end of every sprint and at the end of each recovery period. The rating of perceived exertion (RPE), measured using the Borg Scale (6 to 20) [32], was also recorded at these time points. Breath-by-breath measures of exhaled air were obtained, using the Oxycon Pro (Jaeger, Wuerzburg, Germany) apparatus, during sprint number one, recovery period number one, and sprint and recovery period number three. The exercise bout ended upon completing sprint number four, with a blood sample being obtained immediately, followed by an appetite measure. Thus, the exercise bout lasted a total of 14 min. In the resting trial condition, the participant remained sedentary for an equal amount of time. A 10-min resting exhaled air measurement was made during this time, using the Oxycon Pro apparatus.

On concluding the exercise bout (*t* = 0*), a 2 h rest period began. During this period, the participant again remained sedentary. Blood samples and appetite measures were obtained every 30 min during this time and a resting exhaled air measurement was taken at 60 min post-exercise. At *t* = 120*, the participant was escorted to the research kitchen facility, where they were provided with a buffet lunch meal. The content of the buffet, along with the macronutrient content for each food item, can be found in the Appendix. The participant was instructed that they may consume until satisfied, and were left to do so in isolation. Upon finishing the meal, a final blood sample was obtained and a final appetite measure was recorded.

### 2.5. Measures

Subjective appetite was assessed using the visual analogue scale method (VAS). The four-question, 150 mm-line VAS test for subjective appetite, as adapted from Hill & Blundell [33], was utilised, addressing “hunger,” “fullness,” “desire to eat,” and “expected food intake”. A single VAS test score for appetite was calculated from the four constructs as, hunger score + desire score + expected intake score + (150-fullness score). This single composite score was used for ease of data analysis and presentation, as it has been shown that, with the original six question VAS technique of Hill & Blundell [33], the scores for each question co-vary to a large extent [34] and that the first principle component of the questions is the mean value of the scores [35].

From the resting exhaled air measurement collected at 60 min post-exercise, energy expenditure was calculated. Participants rested in a seated position for a minimum of 30 min prior to sampling gases. Gases were sampled using the Oxycon Pro (Jaeger, Wuerzburg, Germany) apparatus for 10 min. Oxygen consumption and the respiratory exchange ratio were recorded and averaged over the final 5 min of the 10 min sampling period, to calculate an estimate of the energy expenditure for the post-exercise period using the equation of Frayn [36]. 

Post-exercise food intake was assessed using the *ad libitum* buffet meal technique. All food presented (see Appendix A) was pre-weighed. Upon the completion of the meal, all food was re-weighed. Energy intake (kcal) and macronutrient intake (expressed as total grams and percentage of total energy intake derived from each macronutrient) were calculated using Dietplan (version 6.0).

### 2.6. Blood Sampling and Analysis

All blood samples were immediately transferred to disodium EDTA-treated tubes to preserve them for the analysis of hormones. For the measure of GLP-1 and acylated ghrelin concentrations, test tubes were pre-treated with the protease inhibitors DPP IV inhibitor (Millipore, MA, USA—0.01 mL per millilitre of blood) and 4-(2—Aminoethyl) benzenesulfonylfluoride hydrochloride (AEBSF, Alexis Biochemicals, Lausen, Switzerland—0.02 mL of per millilitre of blood). Samples obtained for the measure of glucose were transferred to disodium EDTA-treated tubes that had not undergone further treatment. Blood was centrifuged at 3000 RPM at a temperature of 4 °C for 15 min, to isolate the plasma. Plasma was separated and transferred to sample cups for later analysis. Two sample cups were pre-treated with hydrochloric acid (100 microlitres per millilitre of plasma), to protect acylated ghrelin from degradation. Plasma was stored at a temperature of −70 °C, until the hormone assays were conducted. Plasma glucose (glucose oxidase reagent, Instrumentation Laboratories Company, Monza 338, Milan, Italy) concentration was measured using the ILab 650 Clinical Chemistry System (Instrumentation Laboratories Company, Lexington, MA, USA). Acylated ghrelin and total GLP-1 were measured in duplicate using the ELISA technique (Millipore, MA, USA). All samples were analysed in duplicate, and all samples from a participant were analysed in the same assay. The sensitivity of the ELISA kits was 8 pg·mL^−1^ and 1.5 pg·mL^−1^ for acylated ghrelin and GLP-1, respectively. The intra-assay coefficients of variation were 3.8% and 3.0%, respectively. For all analyses, the values of the quality controls provided by the manufacturers fell within the acceptable range.

### 2.7. Statistical Analysis

All data are presented as mean values ± standard deviation. To assess changes in subjective appetite over time and between the two trial conditions, a 2 × 9 factorial ANOVA with repeated measures was conducted, using the VAS scores. For an analysis of GLP-1 and acylated ghrelin concentrations, 2 × 7 factorial ANOVA with repeated measures were carried out, and for the analysis of glucose concentration, a 2 × 2 factorial ANOVA with repeated measures was conducted. Any significant interactions or main effects were investigated further by carrying out pairwise comparisons, using Bonferroni post-hoc analysis. An attempt was made to control for any variability in the participants’ effort during the exercise bout. On inspection, the covariate of RPE did not significantly correlate with any of the outcome variables, so conducting an ANCOVA was deemed not appropriate. Area-under-the-curve (AUC) was calculated for all profiles plotted, using the trapezoidal method. AUC values were compared using paired-sample T-tests. Energy intake values at the *ad libitum* buffet meal, as well as the macronutrient intake (grams), were compared using paired-sample T-tests. 

The effect sizes of partial eta squared (*η_p_^2^*) were calculated for ANOVA and Cohen’s d (d) were calculated for *t*-tests and post hoc pairwise comparisons. Ninety-five percent confidence intervals (95% CI) are included where relevant. A statistical significance level of *p* < 0.05 was used throughout. All statistical analysis was carried out using the SPSS software programme (SPSS Inc., Chicago, IL, USA).

An a priori power calculation was conducted using the G*Power software. Based on an interpretation of data from a previous study investigating a similar research question [22], and with an alpha level of 0.05 and statistical power of 0.8, a sample size of 12 participants was deemed sufficient to detect a meaningful change in subjective appetite.

## 3. Results

### 3.1. Characteristics of Resting and Exercise Conditions

Table 1 shows the characteristics of REST and EX. The energy expenditure over the entire trial period was significantly higher in the exercise condition (*p* < 0.001), as was the energy expenditure during the exercise bout, compared with the equivalent time point in REST (*p* = 0.001).

### 3.2. Subjective Appetite

**Figure 1:**
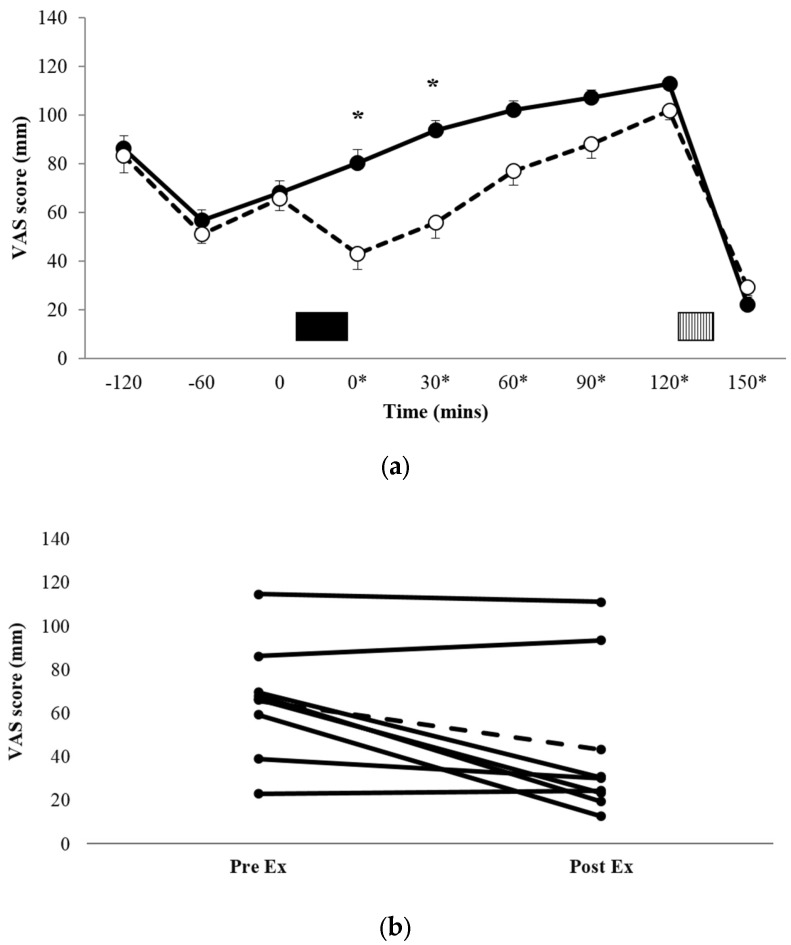
Mean appetite scores (**a**) for REST (●, solid line) and EX (○, dashed line). Solid block represents exercise. Stripe block represents the *ad libitum* test meal. * on x axis labels indicates min post-exercise. Values are mean ± SEM. * = exercise significantly different to rest, *p* < 0.05. Individual appetite responses immediately post exercise (**b**) are also shown for EX. The dashed line represents the mean response.

Figure 1a shows the profiles for subjective appetite scores, and Figure 1b shows the individual appetite scores immediately pre-exercise and immediately post-exercise for each of the eight participants. There was a significant condition x time interaction (*p* = 0.033, *η_p_^2^* = 0.338). Post-hoc pairwise comparisons for between-condition differences demonstrated that appetite scores during EX were significantly lower than REST immediately post exercise (*t* = 0*, 43.0 ± 37.2 mm vs. 80.3 ± 30.2 mm, *p* = 0.017, *d* = 1.101, 95% CI = −65.5–−9.15 mm) and *t* = 30* (55.9 ± 37.7 mm vs. 93.6 ± 24.0 mm, *p* = 0.024, *d* = 1.193, 95% CI = −68.8–−6.57 mm). There was also a trend for a significant difference at *t* = 60* (*p* = 0.073, *d* = 0.884, 95% CI = −3.09–53.1 mm). AUC data for the VAS appetite profiles (Table 2) showed that appetite over the entire trial period was significantly lower in EX versus REST (*p* = 0.047, *d* = 0.764). 

When assessing individual responses (Figure 1b), there appeared to be four large responses (three male, one female), two small responses (both female), and two individuals for whom appetite was unaffected by exercise (one male, one female).

### 3.3. Appetite Regulating Hormones

#### 3.3.1. Acylated Ghrelin 

**Figure 2:**
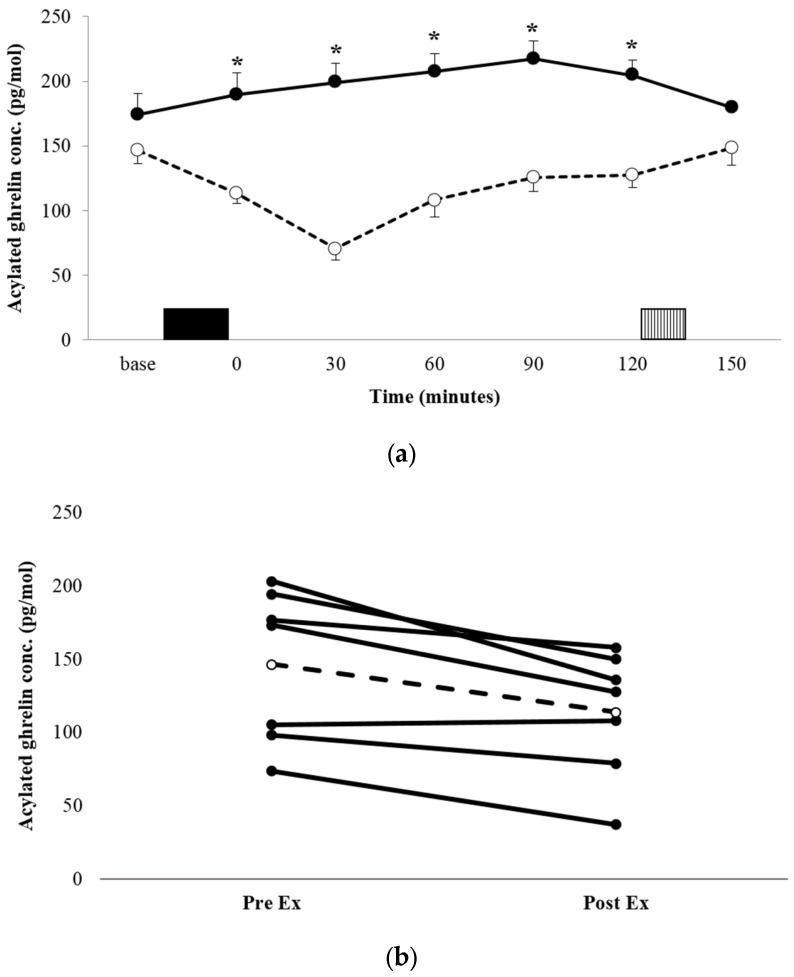
Mean appetite acylated ghrelin concentrations (**a**) for REST (●, solid line) and EX (○, dashed line). Solid block represents exercise. Stripe block represents the *ad libitum* test meal. Values are mean ± SEM. * = exercise significantly different to rest, *p* < 0.05. Individual responses immediately post exercise (**b**) are also shown for EX. The dashed line represents the mean response.

The acylated ghrelin concentration profiles for both conditions are shown in Figure 2a. There was a significant condition x time interaction (*p* = 0.008, *η_p_^2^* = 0.529). Post-hoc analysis for between-condition effects revealed that the acylated ghrelin concentration was significantly lower during the immediate post-exercise period in EX, compared with the equivalent time point in REST (113.4 ± 43.0 pg·mL^−1^ vs. 189.2 ± 91.8 pg·mL^−1^, *p* = 0.03, *d* = 1.057 95% CI = 10.12–141.5 pg·mL^−1^), and remained lower at every time point post-exercise, until the test meal (all *p* < 0.05). AUC was significantly lower in EX compared with REST (*p* = 0.016, *d* = 1.263; Table 2). The decrease in plasma acylated ghrelin was a robust observation, with all but one participant demonstrating a suppression of a similar magnitude (Figure 2b).

#### 3.3.2. GLP-1

**Figure 3:**
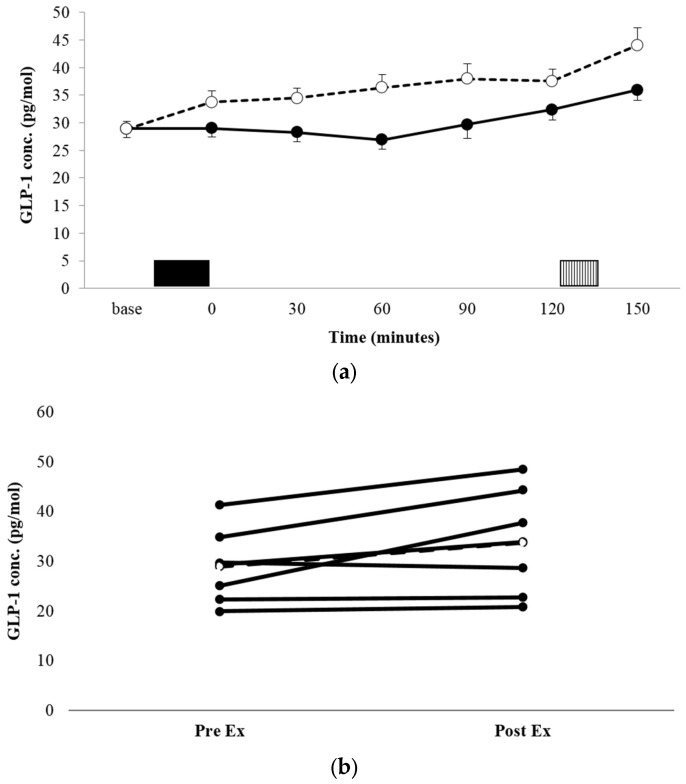
Mean GLP-1 concentrations (**a**) for REST (●, solid line) and EX (○, dashed line). Solid block represents exercise. Stripe block represents the *ad libitum* test meal. Values are mean ± SEM. Individual responses immediately post exercise (**b**) are also shown for EX. The dashed line represents the mean response.

There was no significant condition x time interaction for the GLP-1 concentration (*p* = 0.135, *η_p_^2^* = 0.270, Figure 3a). There was a significant condition main effect, with the mean GLP-1 concentration being significantly higher across the entire trial period in EX vs. REST (36.16 ± 10.97 pg·mL^−1^ vs. 30.16 ± 8.87 pg·mL^−1^, *p* = 0.002, *η_p_^2^* = 0.825). Comparisons of AUC for GLP-1 concentration profiles (Table 2) showed that the area was significantly greater in EX, compared with REST (*p* = 0.001, *d* = 2.163). Individual responses to exercise suggest three large responses (all male), one small response (male), and three individuals for whom GLP-1 was unaffected by exercise (all female).

### 3.4. Food Intake and Energy Balance

**Figure 4:**
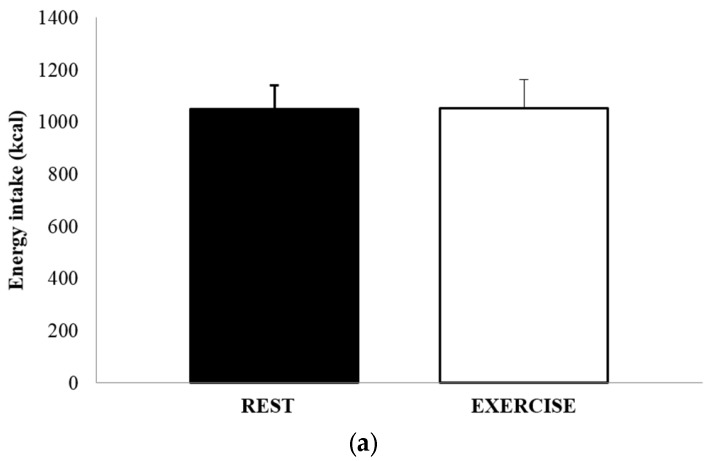

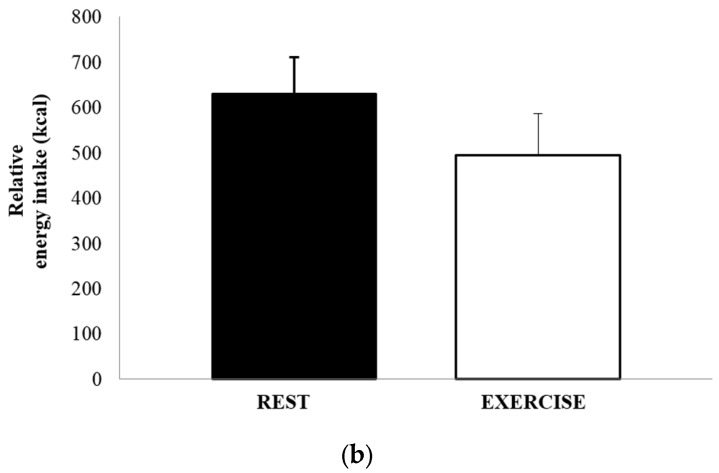
Absolute energy intake (**a**) and relative energy intake (**b**), for REST (solid black bar) and EX (empty bar). Values are mean ± SEM.

The mean absolute energy intake and mean relative energy intake for both REST and EX are shown in Figure 4a,b. There was no significant difference in either energy intake (1051 ± 502 kcal vs. 1052 ± 615 kcal, *p* = 0.991, *d* = 0.002), or relative energy intake (630 ± 458 kcal vs. 494 ± 514 kcal, *p* = 0.264, *d* = 0.279). The total amount of food consumed, in grams, and the macronutrient content of the food consumed, expressed as both absolute intake (grams) and percentage of total energy, are shown in Table 3. There were no differences between REST and EX for any of these values. 

## 4. Discussion

Just 4 × 30 s of maximal effort sprint cycling was sufficient to elicit a transient suppression of subjective appetite, compared with a resting condition. While other protocols of supramaximal high-intensity intermittent exercise have suppressed appetite in healthy-weight individuals [22,23], and submaximal intermittent exercise has resulted in small, non-significant reductions in appetite in overweight males [28,29], the present study demonstrates the lowest total volume of exercise to elicit an appetite suppression in overweight individuals. This occurred despite a very low duration and energy cost of the exercise; the mean energy expenditure for the exercise bout (including the recovery period between repetitions) was estimated to be 152 kcal and the total duration of the bout was 14 min, with a total of only 2 min worth of intense exercise. A lower duration bout of 10 min of cycling incorporating two 20 s “all out” sprints, failed to suppress appetite in healthy-weight individuals [24], which suggests that there may be a duration or energy expenditure “threshold” for reducing appetite sensations, but that this lies below that of the present study. 

As is often the case with exercise-induced appetite suppression, this effect was short-lived, although there was a trend for a lower appetite score 60 min post-exercise. The absence of a difference in subjective appetite after 60 min was allied with no difference in food intake at the *ad libitum* test meal at *t* = 120*. The mean energy intake was very similar for the two conditions, differing by only 18 kcal (1.7%). When accounting for the energy cost of both rest and exercise, the relative energy intake, despite being 20% lower in the exercise trial, was not significantly different between conditions. As food intake was not assessed beyond lunch in the present pilot investigation, any delayed effect of exercise on intake could not be assessed. This finding—a transient suppression of appetite, with no immediate consequent influence on food intake—is in agreement with the findings of previous investigations into the effect of sprint interval exercise on appetite in normal weight individuals [22,23], but only partly supports the findings of previous studies of appetite responses to high-intensity intermittent exercise in overweight and obese individuals [27,28,29]. In these incidences, small, non-significant decreases [28,29] and increases [27] in subjective appetite have been observed. This discrepancy is possibly due to the differing intensity of the exercise bouts, with a very strenuous protocol of 30 s “all-out” maximal effort cycling undertaken in the current study, as opposed to the submaximal effort high-intensity cycling of the aforementioned studies. 

The lack of an effect of the exercise bout on food intake may be due to the 2-h period between the cessation of exercise and the *ad libitum* meal. Reductions in food intake after exercise are rarely observed when measured ≥60 min post exercise [4,37,38,39,40], so this finding is not surprising. It would be interesting to assess the differences in eating behaviour when allowing *ad libitum* feeding from the moment of exercise cessation, including not only the amount of food consumed, but also the timing of feeding. However, as the primary interest of the present study was to address time-course changes in subjective appetite and appetite-associated hormones, such an approach was not appropriate. Given the observed suppression of appetite, food intake and eating latency may prove altered in the immediate post-exercise period. Further, when considering the very low volume of the exercise performed in the present study, such bouts may prove a time-efficient means of altering appetite to reduce food intake or delay the initiation of feed, if administered at strategic time-points of the day, such as prior to meals or at times when individuals are prone to snacking. While at this stage speculative, the potential for such responses to “exercise snacking” warrant further investigation.

The suppression of subjective appetite was allied with changes in appetite-associated hormones, in favour of an anorexigenic state. Acylated ghrelin was suppressed immediately after exercise and continued to fall, with the lowest concentration observed 30 min post-exercise. At this point, the plasma concentration was 64% lower than the corresponding time point in the resting trial and had fallen 53% from the baseline. While decreases in acylated ghrelin are usually transient, after both continuous [1,2,37,38] and interval exercise [28,29], the concentrations remained lower than those of the resting trial for the entire two-hour post-exercise period, until the test meal. A similar enduring suppression has been observed after 6 × 30 s of “all out” cycling in healthy-weight, active individuals [22], with acylated ghrelin remaining suppressed until a test meal administered 45 min post-exercise, and calculated AUC remaining lower than that of a control condition for a prolonged 6-h post-exercise period. This finding suggests that acylated ghrelin may be yet more responsive to supramaximal exercise in overweight and obese individuals, evidenced by the prolonged suppression observed in the present study. However, the dissociation with appetite and food intake does question the role of acylated ghrelin in regulating appetite and eating behavior in the post-exercise period.

GLP-1 proved less responsive to exercise. Plasma concentrations increased non-significantly with exercise (by 17%), resulting in values being 15% higher in the exercise condition, versus rest. Interestingly, the GLP-1 concentration continued to increase steadily post-exercise, peaking at *t* = 90, while the concentration in the resting condition stayed more constant until *t* = 60, before increasing steadily over the final hour before the test meal. This led to a significantly greater AUC for the entire trial period in the exercise condition, allied with the main effect of the condition. Existing literature regarding the effect of exercise on the plasma GLP-1 concentration is limited. Martins et al. [5] witnessed a significant increase in GLP-1 after intermittent cycling exercise at 65% estimated maximal heart rate (3 × 17 min with 3 min recovery), in healthy, normal-weight individuals, as well as after moderate-intensity continuous and high-intensity intermittent exercise in overweight and obese individuals [25]. In both cases, the increase was modest and transient, unlike the sustained increase observed in the present study. Ueda et al. showed sustained, but modest, significant increases in GLP-1 concentrations after both moderate and high intensity exercise, in both lean and obese individuals [6,7]. These data, allied with that of the present study, would suggest that the GLP-1 response to high-intensity intermittent exercise is similar to that observed with longer duration, continuous exercise of a lower intensity in the overweight and obese.

Interestingly, when inspecting the individual responses of GLP-1 to exercise, there appears a difference between males and females. All four males exhibited immediate post-exercise increases in GLP-1 concentrations of between 15% and 50%, while the three females did not exhibit post-exercise changes. A previous investigation has provided some evidence that an attenuation of the GLP-1 response to a glucose load in overweight, prediabetic, and diabetic individuals, compared with those with normal glucose tolerance, is more pronounced in females [41]. However, recent studies would indicate no sex differences in acute appetite and appetite-associated hormone responses to exercise in healthy-weight individuals [25,42,43].

Nonetheless, the current findings do suggest there may be a gender difference in GLP-1 responses to exercise in overweight individuals, with such a difference warranting further study.

It is acknowledged that a limitation of a pilot study of this nature is a small sample. This may have resulted in insufficient power to detect statistically significant differences, leading to a type II error. As the effect size is independent of the sample size, these values were also included. A post hoc statistical power calculation for the immediate post-exercise appetite response revealed a calculated power of 0.875. One difference exhibiting a large effect—the subjective appetite score at 60 min post-exercise in REST and EX—failed to show statistical significance. A post-hoc power calculation indicated a statistical power for this pairwise comparison, of 0.727. As such, the lack of a statistically significant difference at 60 min post-exercise may be a type II error, suggesting that a more enduring appetite suppression was experienced. Post-hoc power calculations for the ANOVA tests for acylated ghrelin and GLP-1 yielded powers of 0.999 and 0.882, respectively. Further, small sample sizes can result in a type I error if differences in the mean values are driven by very large responses in just one or two participants. However, figures illustrating individual responses in subjective appetite, acylated ghrelin, and GLP-1 demonstrate that this is not the case. Therefore, while the statistical power was low, the validity of the findings of this pilot study appear sound and can be used to inform further investigation. 

A further limitation is the lack of acylated ghrelin and GLP-1 measures at the baseline, prior to the breakfast meal. However, the key period of interest for the present study was the periods immediately prior to, during, and after exercise. Measures prior to this period would have resulted in a greater financial cost and would have required a prolonged period for the participant to have the cannula in the antecubital vein. Hence, the decision was made to obtain the first measure of appetite-associated hormones immediately prior to exercise, to the detriment of assessing early-morning hormone concentrations.

## 5. Conclusions 

Very low volume sprint interval exercise of just 4 × 30 s transiently suppressed appetite in overweight individuals. Appetite suppression was allied with an enduring decrease in the plasma concentration of acylated ghrelin and a modest increase in GLP-1, which may have been restricted to males. However, the relationships between changes in appetite-associated hormones and both subjective appetite and food intake—which was unaffected two-hours post-exercise—were weak, questioning their role in post-exercise appetite regulation. Nonetheless, these findings suggest that the potential for micro bouts of “exercise snacks” to influence appetite and very short-term eating behavior warrants further investigation.

## Figures and Tables

**Table 1:** Characteristics of the exercise and rest conditions.

Condition	Estimated EE for Trial (kcal)	Estimated EE for Bout (kcal)	HR_average_ (Beats·min^−1^)	RPE	Peak Power (Watts)	Mean Power (Watts)	Work (Joules)
Rest	445 ± 107	31.0 ± 7.49	-	-	-	-	-
Exercise	582 ± 112 *	158 ± 29.0 *	131 ± 21.2	17 ± 2	767 ± 278	454 ± 123	13792 ± 6693

Values are mean ± SD. * = significantly different to Rest, *p* < 0.01.

**Table 2:** Area-under-the-curve values for subjective appetite and plasma concentrations of acylated ghrelin and GLP-1.

	Rest	Exercise
**Appetite**	675 ± 158 mm·290 min^−1^	539 ± 196 mm·290 min^−1^ *
**Acylated ghrelin**	1045 ± 568 pg·mL^−1^·155 min^−1^	606 ± 372 pg·mL^−1^·155 min^−1^ *
**GLP-1**	156 ± 80 pg·mL^−1^·155 min^−1^	190 ± 98 pg·mL^−1^·155 min^−1^ *

Values are mean ± SD. * = significantly different to Rest, *p* < 0.05.

**Table 3:** Food intake at the *ad libitum* test meal.

	Rest	Exercise
**Volume (grams)**	865 ± 445	733 ± 345
**CHO (g)**	154 ± 59.4	154 ± 99
**% E CHO**	61.8 ± 10.4	58.7 ± 11.1
**Fat (g)**	32.1 ± 24.8	33.5 ± 23.0
**% E fat**	24.2 ± 11.0	27.1 ± 11.2
**PRO (g)**	38.8 ± 24.5	37.0 ± 16.8
**% E PRO**	14.8 ± 5.2	15.4 ± 4.8

CHO = carbohydrate. % E = percentage of total energy intake. Values are mean ± SD.
